# Efficacy and safety of rucaparib in previously treated, locally advanced or metastatic urothelial carcinoma from a phase 2, open-label trial (ATLAS)

**DOI:** 10.1186/s12885-021-08085-z

**Published:** 2021-05-24

**Authors:** P. Grivas, Y. Loriot, R. Morales-Barrera, M. Y. Teo, Y. Zakharia, S. Feyerabend, N. J. Vogelzang, E. Grande, N. Adra, A. Alva, A. Necchi, A. Rodriguez-Vida, S. Gupta, D. H. Josephs, S. Srinivas, K. Wride, D. Thomas, A. Simmons, A. Loehr, R. L. Dusek, D. Nepert, S. Chowdhury

**Affiliations:** 1 Department of Medicine, Division of Medical Oncology, University of Washington, Seattle, WA 98109 USA; 2 Clinical Research Division, Fred Hutchinson Cancer Research Center, Seattle, WA 98109 USA; 3 Seattle Cancer Care Alliance, 1144 Eastlake Avenue E, LG- 465, Seattle, WA 98109 USA; 4 Department of Medicine, Gustave Roussy Cancer Campus, INSERM U981, Université Paris-Saclay, 39 Rue Camille Desmoulins, 94800 Villejuif, France; 5 Passeig Vall d’Hebron 119-129, 08035, Barcelona, Spain; 6 Department of Medicine, Memorial Sloan Kettering Cancer Center, 1275 York Avenue, New York, NY 10065 USA; 7 Division of Hematology, Oncology, and Blood and Marrow Transplant, University of Iowa and Holden Comprehensive Cancer Center, 200 Hawkins Drive, Iowa City, IA 52242 USA; 8 Studienpraxis Urologie, Steinengrabenstraße 17, 72622 Nürtingen, Germany; 9 Division of Hematology/Oncology, Comprehensive Cancer Centers of Nevada, 3730 S Eastern Avenue, Las Vegas, NV 89169 USA; 10 Department of Medical Oncology, MD Anderson Cancer Center, Calle de Arturo Soria, 270 28033 Madrid, Spain; 11 Department of Medicine, Indiana University Simon Cancer Center, 535 Barnhill Drive, Indianapolis, IN 46202 USA; 12 Department of Internal Medicine, University of Michigan Comprehensive Cancer Center, 1500 E Medical Center Drive, Ann Arbor, MI 48109 USA; 13 Department of Medical Oncology, Fondazione IRCCS Istituto Nazionale dei Tumori, Via Giacomo Venezian, 1, 20133 Milan, Italy; 14 Medical Oncology Department, Hospital del Mar, Passeig Maritim 25-29, 08003 Barcelona, Spain; 15 Division of Medical Oncology, Huntsman Cancer Institute, University of Utah, 1950 Circle of Hope, Salt Lake City, UT 84112 USA; 16 Department of Medical Oncology, Guy’s and St. Thomas’ NHS Foundation Trust, Great Maze Pond, London, SE1 9RT UK; 17 Division of Medical Oncology, Stanford University School of Medicine, 875 Blake Wilbur Drive, Stanford, CA 94305 USA; 18 Clovis Oncology, Inc., 5500 Flatiron Parkway, Boulder, CO 80301 USA; 19 Department of Medical Oncology, Guy’s and St. Thomas’ NHS Foundation Trust & Sarah Cannon Research Institute, Great Maze Pond, London, SE1 9RT UK

**Keywords:** Bladder cancer, Urothelial carcinoma, PARP inhibitor, Rucaparib, Genomic biomarkers

## Abstract

**Background:**

ATLAS evaluated the efficacy and safety of the PARP inhibitor rucaparib in patients with previously treated locally advanced/unresectable or metastatic urothelial carcinoma (UC).

**Methods:**

Patients with UC were enrolled independent of tumor homologous recombination deficiency (HRD) status and received rucaparib 600 mg BID. The primary endpoint was investigator-assessed objective response rate (RECIST v1.1) in the intent-to-treat and HRD-positive (loss of genome-wide heterozygosity ≥10%) populations. Key secondary endpoints were progression-free survival (PFS) and safety. Disease control rate (DCR) was defined post-hoc as the proportion of patients with a confirmed complete or partial response (PR), or stable disease lasting ≥16 weeks.

**Results:**

Of 97 enrolled patients, 20 (20.6%) were HRD-positive, 30 (30.9%) HRD-negative, and 47 (48.5%) HRD-indeterminate. Among 95 evaluable patients, there were no confirmed responses. However, reductions in the sum of target lesions were observed, including 6 (6.3%) patients with unconfirmed PR. DCR was 11.6%; median PFS was 1.8 months (95% CI, 1.6–1.9). No relationship was observed between HRD status and efficacy endpoints. Median treatment duration was 1.8 months (range, 0.1–10.1). Most frequent any-grade treatment-emergent adverse events were asthenia/fatigue (57.7%), nausea (42.3%), and anemia (36.1%). Of 64 patients with data from tumor tissue samples, 10 (15.6%) had a deleterious alteration in a DNA damage repair pathway gene, including four with a deleterious *BRCA1* or *BRCA2* alteration.

**Conclusions:**

Rucaparib did not show significant activity in unselected patients with advanced UC regardless of HRD status. The safety profile was consistent with that observed in patients with ovarian or prostate cancer.

**Trial registration:**

This trial was registered in ClinicalTrials.gov (NCT03397394). Date of registration: 12 January 2018. This trial was registered in EudraCT (2017–004166-10).

**Supplementary Information:**

The online version contains supplementary material available at 10.1186/s12885-021-08085-z.

## Background

Bladder cancer is one of the most common cancer types [1], with urothelial carcinoma (UC) accounting for > 90% of the cases [2]. In 2018, there were approximately 549,000 estimated new bladder cancer cases and 200,000 deaths worldwide [3]. The prognosis for advanced disease is poor, with a 5-year overall survival (OS) rate of only 5% in those diagnosed with distant metastases [4].

Platinum-based chemotherapy (PBC) has been the standard upfront treatment for patients with locally advanced/unresectable or metastatic UC [5]. Single chemotherapy agents, such as taxanes, gemcitabine, pemetrexed, or vinflunine (European Union only), are used in patients whose disease progressed on or after a PBC [6, 7]. However, these second-line treatments are associated with modest objective response rates (ORRs, 14–32%), with a median OS of 7–8 months [6, 8]. In the post-platinum setting, single-agent immune checkpoint inhibitors (ICI) have provided clinical benefit in patients with platinum-refractory UC (ORRs, 13–21%) [9, 10]. Notably, in the Keynote-045 trial, pembrolizumab demonstrated an OS advantage and a more favorable toxicity profile versus second-line chemotherapy [10]. However, many patients continue to have early progression and/or toxicity with current therapies, emphasizing the need for additional therapeutic options and research into validated biomarkers that can identify patients who are more likely to derive enduring therapeutic benefit [11, 12].

Rucaparib is a potent, oral, small-molecule inhibitor of poly(ADP-ribose) polymerase (PARP) enzymes [13], which play a role in DNA damage repair (DDR). Rucaparib recently received accelerated approval from the US Food and Drug Administration (FDA) as single-agent therapy for patients with metastatic castration-resistant prostate cancer and is approved in the United States and the European Union for treatment or maintenance treatment of patients with recurrent ovarian cancer [14, 15]. Although data on PARP inhibitors in UC have been limited [16, 17], the following evidence suggests that a subset of urothelial tumors may be susceptible to PARP inhibition. Alterations in DDR genes (e.g. *BRCA1* or *BRCA2* [*BRCA*]) have been observed in 11% of patients with UC [18], and approximately 60% of patients with UC exhibit homologous recombination deficiency (HRD; e.g. high genome-wide loss of heterozygosity [LOH] or deleterious DDR gene alteration). Moreover, patients with UC are usually sensitive to PBC [19, 20], and platinum sensitivity in other indications has been associated with response to PARP inhibitors [21]. In clinical trials, the benefit of rucaparib was observed in both HRD-positive and HRD-negative ovarian tumors [22–25]. Based on these data, we hypothesized that rucaparib monotherapy could potentially be safe and effective in patients with locally advanced/unresectable or metastatic UC independent of tumor HRD status. Unselected enrollment of patients would also facilitate feasible accrual of patients with DDR gene alterations that are uncommon in UC, while allowing the study to be a priori powered to assess the efficacy of rucaparib in patients with HRD-positive tumors.

Here we report the final efficacy and safety results from the ATLAS study, which evaluated rucaparib in patients with locally advanced/unresectable or metastatic UC. In addition, we report the tumor genomic features of patients enrolled in this study.

## Methods

### Study design

ATLAS (NCT03397394; EudraCT 2017–004166-10) was an international, open-label, phase 2 study that evaluated the efficacy and safety of single-agent rucaparib for patients with locally advanced/unresectable or metastatic UC previously treated with one or two anticancer systemic regimens. The study was approved by national or local institutional review boards and was performed in accordance with the Declaration of Helsinki and Good Clinical Practice Guidelines of the International Council for Harmonisation. Patients provided written informed consent before participation.

### Patients

Eligible patients were aged ≥18 years, had locally advanced/unresectable or metastatic UC with measurable disease per Response Evaluation Criteria In Solid Tumors version 1.1 (RECIST v1.1), and had confirmed radiographic progression following one or two prior treatment regimens (e.g. cisplatin- or carboplatin-containing chemotherapy, ICI, and/or investigative agent). No more than one prior PBC was permitted for advanced disease. Patients who had never received platinum must have been ineligible for or refused cisplatin at the time of study entry. Patients had an Eastern Cooperative Oncology Group (ECOG) performance status of 0 or 1, and adequate organ function. Patients were enrolled independent of tumor HRD status as defined by genome-wide LOH [26]. However, tumor tissue collected prior to treatment was required for molecular profiling. Patients with prior PARP inhibitor treatment were excluded. Patients provided written informed consent before participating in the study. Additional inclusion criteria are described in the Supplementary Methods.

### Procedures

Tumor tissue samples obtained within 28 days prior to initiating rucaparib, or within 6 months if no intervening therapy, were mandatory at baseline. If available, archival tumor samples from earlier time points were also collected. Tumor HRD status and DDR gene alterations were identified using the DX1 next-generation sequencing (NGS) assay from Foundation Medicine, Inc. (Cambridge, MA, USA) [25]. In the ARIEL2 study of rucaparib in ovarian cancer, sensitivity to platinum-containing chemotherapy and high genomic LOH (thought to be a biomarker of HRD) were associated with rucaparib benefit in ovarian cancer [25, 27]. To prospectively define HRD-positive status in the ATLAS trial, we analyzed a subset of patients from The Cancer Genome Atlas Urothelial Bladder Carcinoma (TCGA-BLCA) dataset [26] who were treated with platinum-based chemotherapy. Survival benefit was analyzed in platinum-treated patients at all possible genomic LOH cut-offs. In patients with genomic LOH ≥10%, platinum-based chemotherapy showed a statistically significant survival benefit compared to patients with genomic LOH < 10%. This cut point resulted in a favorable hazard ratio, *p*-value, sensitivity, and specificity. Therefore, we defined HRD-positive status as genomic LOH ≥10% for the ATLAS study.

Details of the TCGA-BLCA dataset analyses are described in the Supplementary Methods. Blood samples were also collected at baseline and various time points for circulating tumor DNA (ctDNA) analysis.

Patients received oral rucaparib 600 mg twice daily until confirmed radiographic disease progression by investigator assessment, unacceptable toxicity, or other reason for discontinuation. Dose reduction criteria are described in the Supplementary Methods.

Disease assessments were conducted by the investigator based on clinical examination and appropriate imaging technique using RECIST v1.1 [28]. The first postbaseline radiographic scan was to be performed at 8 weeks (±7 days). If a patient had signs of progression prior to the initial radiographic tumor assessment, the treating investigator could choose to perform imaging at an earlier time point. Assessments were conducted every 8 weeks for up to 18 months, then every 12 weeks thereafter, including for patients who discontinued treatment for reasons other than disease progression. Radiographic tumor assessments were continued until confirmed radiographic disease progression, loss to follow-up, withdrawal from the study, study closure, or initiation of subsequent treatment.

Patients were monitored for adverse events (AEs), serious AEs, and AEs of special interest during study participation and until 28 days after the last dose of rucaparib. AEs and laboratory abnormalities were graded according to the National Cancer Institute’s Common Terminology Criteria for Adverse Events grading system version 4.03 or later.

Plasma samples were collected for trough level pharmacokinetic (PK) analysis of rucaparib 1 h before the morning dose on days 29, 57, and 85.

### Outcomes

The primary endpoint was ORR per investigator assessment using RECIST v1.1 in the intent-to-treat (ITT) population (all patients who received ≥1 dose of rucaparib) and in patients with HRD-positive tumors. Key secondary endpoints included duration of response, progression-free survival (PFS), safety and tolerability, and the steady-state PK of rucaparib. Disease control rate (DCR), defined post-hoc as the proportion of patients with a confirmed complete or partial response, or stable disease lasting ≥16 weeks, was also assessed. Exploratory endpoints included assessing tumor tissue- and blood-based biomarkers that correlate with response to rucaparib and molecular changes over time in tumor samples. Safety was assessed by monitoring AEs and vital signs, physical examination, and laboratory testing.

### Statistical analysis

ATLAS was designed to enroll approximately 200 patients. Details of sample size calculation are described in the Supplementary Methods. An adaptive study design was used, in which interim efficacy and safety analyses were performed to determine whether to continue enrollment. Two interim efficacy analyses were planned after efficacy data (defined as a documented objective response per RECIST v1.1, disease progression, or at least 4 months of disease assessments) were available for 60 and 120 patients. Enrollment was halted following the data monitoring committee’s review for the first interim analysis. We present the final results from the database lock date of February 20, 2020.

Efficacy analyses were performed using the ITT population and subgroups based on tumor HRD status (HRD-positive [genomic LOH ≥10%], HRD-negative [genomic LOH < 10%], and HRD-indeterminate). ORR and safety endpoints are summarized using descriptive statistics and PFS using Kaplan-Meier methodology. PK parameters are summarized using descriptive statistics.

## Results

### Patients

**Fig. 1:**
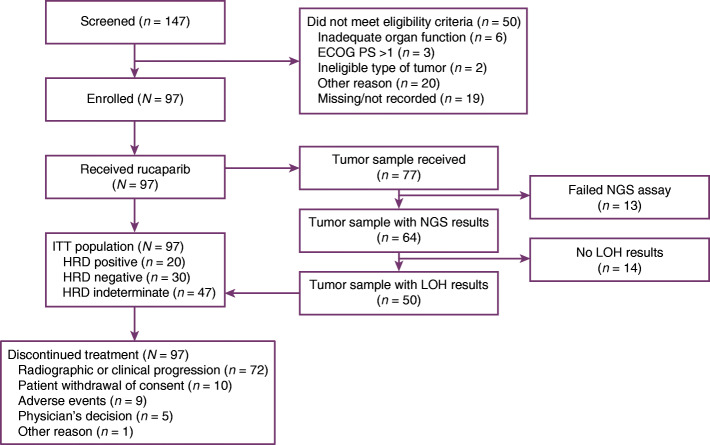
Trial profile. *ECOG PS* Eastern Cooperative Oncology Group performance status; *HRD* homologous recombination deficiency; *ITT* intent-to-treat; *LOH* loss of heterozygosity; *NGS* next-generation sequencing

**Fig. 2:**
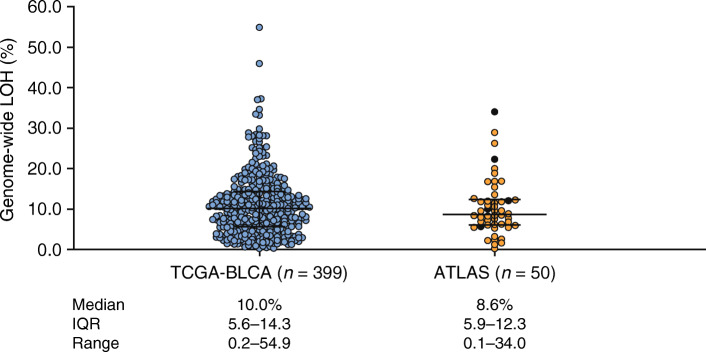
Genome-wide LOH in TCGA-BLCA dataset and tumor tissue samples. Each circle represents a tissue sample, and the bars represent the median and interquartile range. Black circles in the ATLAS dataset highlight samples with deleterious alterations in DDR pathway genes *BRCA1*, *BRCA2*, *PALB2*, or *RAD51C. DDR* DNA damage response; *IQR* interquartile range; *LOH* loss of heterozygosity; *TCGA-BLCA* The Cancer Genome Atlas Urothelial Bladder Carcinoma

Ninety-seven patients were enrolled between June 1, 2018, and April 9, 2019, across 70 sites in six countries (France, Germany, Italy, Spain, the United Kingdom, and the United States; Fig. 1). Baseline demographics and disease characteristics of patients are provided in Table 1. Overall, 44/97 (45.4%) patients had received two prior therapies for advanced disease; 93/97 (95.9%) patients had received prior PBC, 71/97 (73.2%) patients prior ICI, and 67/97 (69.1%) patients both a PBC and an ICI (separately or combined). Of 97 patients, 77 (79.4%) provided tumor-containing tissue samples that underwent genomic testing. The majority (68/77 [88.3%]) of the samples in the analyzed dataset were obtained within 6 months of initiating rucaparib with no intervening therapy. LOH was determined for 50/97 patients (51.5%). Of 97 patients, 20 (20.6%) had HRD-positive tumors (LOH ≥10%), 30 (30.9%) HRD-negative tumors (LOH < 10%), and 47 (48.5%) indeterminate HRD status (i.e. the tumor sample was not provided or the sample quantity/quality was insufficient). The median genome-wide LOH was 8.6% (interquartile range, 5.9–12.3), consistent with data from the TCGA-BLCA dataset (10.0% [interquartile range, 5.6–14.3]; Fig. 2).

**Table 1:** Baseline demographics, disease characteristics, and prior therapies

Characteristic	HRD subgroup^**a**^			Overall (N = 97)
	Positive (*n* = 20)	Negative (*n* = 30)	Indeterminate^b^ (*n* = 47)	
Age, median (range), y	71 (39–87)	66 (47–85)	66 (50–85)	66 (39–87)
Sex, *n* (%)				
Male	11 (55.0)	27 (90.0)	38 (80.9)	76 (78.4)
Female	9 (45.0)	3 (10.0)	9 (19.1)	21 (21.6)
Race, *n* (%)				
White	18 (90.0)	24 (80.0)	32 (68.1)	74 (76.3)
Black or African American	0	2 (6.7)	1 (2.1)	3 (3.1)
Other	0	1 (3.3)	2 (4.3)	3 (3.1)
Unknown	2 (10.0)	3 (10.0)	12 (25.5)	17 (17.5)
ECOG PS, *n* (%)				
0	6 (30.0)	9 (30.0)	14 (29.8)	29 (29.9)
1	14 (70.0)	20 (66.7)	32 (68.1)	66 (68.0)
2^c^	0	1 (3.3)	1 (2.1)	2 (2.1)
Histology, *n* (%)				
Urothelial	14 (70.0)	25 (83.3)	30 (63.8)	69 (71.1)
Urothelial with variant	5 (25.0)	2 (6.7)	7 (14.9)	14 (14.4)
Unknown	1 (5.0)	3 (10.0)	10 (21.3)	14 (14.4)
Tumor location in bladder, *n* (%)				
Lower tract	17 (85.0)	22 (73.3)	37 (78.7)	76 (78.4)
Upper tract	3 (15.0)	8 (26.7)	10 (21.3)	21 (21.6)
No. of prior therapies, *n* (%)				
1	11 (55.0)	16 (53.3)	26 (55.3)	53 (54.6)
2	9 (45.0)	14 (46.7)	21 (44.7)	44 (45.4)
Prior therapies, *n* (%)^d^				
Cisplatin-based chemotherapy	13 (65.0)	20 (66.7)	26 (55.3)	59 (60.8)
Carboplatin-based chemotherapy	5 (25.0)	8 (26.7)	21 (44.7)	34 (35.1)
Immune checkpoint inhibitor	14 (70.0)	23 (76.7)	34 (72.3)	71 (73.2)
Platinum-based chemotherapy and immune checkpoint inhibitor	12 (60.0)	21 (70.0)	34 (72.3)	67 (69.1)
Cystectomy/nephroureterectomy	8 (40.0)	17 (56.7)	22 (46.8)	47 (48.5)
Time from prior systemic therapy, *n* (%)				
<3 months	15 (75.0)	18 (60.0)	27 (57.4)	60 (61.9)
≥3 months	5 (25.0)	12 (40.0)	20 (42.6)	37 (38.1)
De novo metastases, *n* (%)	12 (60.0)	6 (20.0)	12 (25.5)	30 (30.9)
Site of metastases, *n* (%)^d^				
Nodal metastases	3 (15.0)	7 (23.3)	14 (29.8)	24 (24.7)
Visceral metastases	9 (45.0)	20 (66.7)	23 (48.9)	52 (53.6)
Liver metastases	9 (45.0)	12 (40.0)	14 (29.8)	35 (36.1)
No. of Bellmunt risk factors, *n* (%)^e^				
0	3 (15.0)	6 (20.0)	8 (17.0)	17 (17.5)
1	9 (45.0)	10 (33.3)	23 (48.9)	42 (43.3)
2	7 (35.0)	11 (36.7)	14 (29.8)	32 (33.0)
3	1 (5.0)	3 (10.0)	2 (4.3)	6 (6.2)

*ECOG PS* Eastern Cooperative Oncology Group performance status; *HRD* homologous recombination deficiency; *LOH* loss of heterozygosity.

Data cutoff: February 20, 2020.

^a^ Based on ≥10% genomic LOH cutoff.

^b^ Tumor sample was either not received or not evaluable for percentage of genomic LOH because of insufficient tissue volume, low tumor content, inadequate DNA extraction, or the sample did not meet quality control metrics resulting in reduced sequencing specificity.

^c^ Patients had an ECOG PS score of 1 at screening but were classified with an ECOG PS score of 2 at baseline.

^d^ Categories are not mutually exclusive.

^e^ Bellmunt risk factors were an ECOG PS score >0, a hemoglobin level <10 g/dL, and presence of liver metastases [29].

By February 20, 2020, all patients had discontinued treatment, primarily due to radiographic or clinical disease progression (72/97 [74.2%]). The remaining patients discontinued due to withdrawal of consent (10/97 [10.3%]), AEs (9/97 [9.3%]), physician decision (5/97 [5.2%]), or other reason (discontinued based on new information about the effectiveness of rucaparib; 1/97 [1.0%]; Fig. 1).

After discontinuation of rucaparib, subsequent anticancer therapy was administered to 12/97 (12.4%) patients. The most frequently administered subsequent therapies were docetaxel, paclitaxel, pembrolizumab, and vinflunine, each of which was administered to 2/12 (16.7%) patients.

### Efficacy outcomes

**Fig. 3:**
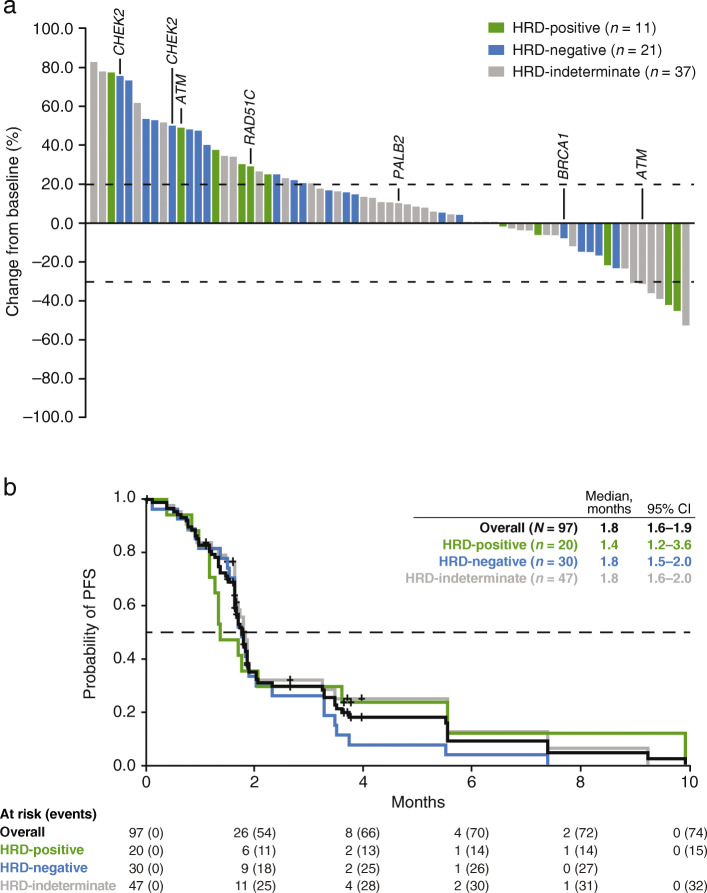
Efficacy outcomes. Investigator-assessed best response in target lesions per RECIST v1.1 in the ITT population (**a**) and Kaplan-Meier estimates of progression-free survival as assessed by the investigator in the overall ITT population and HRD subgroups (**b**). Data cutoff: February 20, 2020. The ITT population only includes patients with measurable disease at baseline and one or more postbaseline tumor assessment (*n* = 69); each bar represents data from a single patient with 0% change from baseline shown as 0.5% for visual clarity; patients with a deleterious mutation in DDR pathway genes *CHEK2, ATM*, *RAD51C*, *PALB2*, and *BRCA1* are indicated. *HRD* homologous recombination deficiency; *ITT* intent-to-treat; *PFS* progression-free survival; *RECIST v1.1* Response Evaluation Criteria in Solid Tumors version 1.1

Of 97 patients enrolled, 95 had measurable disease at baseline. There were no confirmed investigator-assessed objective responses (0%; 95% CI, 0–3.8%) in the overall population or the HRD-positive subgroup. However, reductions in tumor size were observed in a number of patients, including 6/95 (6.3%) patients who had a PR that was not confirmed on subsequent tumor assessment (Fig. 3a). Tumor size reductions were observed in target lesions in lung, kidney, liver, lymph nodes (multiple anatomic sites), and peritoneal metastases, in addition to primary bladder tumors. Among these six patients, two were HRD-positive and four were HRD-indeterminate. Four of the six patients had received ICI as part of their last treatment regimen prior to rucaparib. In addition, 22/95 (23.2%) patients had a best overall response of SD, including a patient in the HRD-indeterminate subgroup with a heterozygous *ATM* alteration who had SD lasting 32 weeks. A similar proportion of patients across the HRD subgroups had a best overall response of SD or better (HRD-positive, 4/19 [21.1%]; HRD-negative, 7/29 [24.1%]; HRD-indeterminate, 17/47 [36.2%]).

The DCR in the overall population was 11.6% (11/95): 15.8% (3/19) in the HRD-positive subgroup, 6.9% (2/29) in the HRD-negative subgroup, and 12.8% (6/47) in the HRD-indeterminate subgroup.

Median PFS was 1.8 months (95% CI, 1.6–1.9) in the ITT population, and was similar across HRD subgroups (Fig. 3b).

### Safety

The safety population included 97 patients who received ≥1 dose of rucaparib. The overall median (range) treatment duration was 1.8 months (0.1–10.1), and median (range) follow-up duration was 2.7 months (0.5–11.1).

A treatment-emergent AE (TEAE) of any grade was reported in 95/97 (97.9%) patients, the most frequent of which (≥25% of patients) were asthenia/fatigue, nausea, anemia, and decreased appetite (Table 2). Furthermore, 71/97 (73.2%) patients had a grade ≥3 TEAE; anemia and thrombocytopenia were the most frequent (Table 2). A total of 76/97 (78.4%) patients reported having at least one treatment-related AE (Supplementary Table S1). While myelodysplastic syndrome and acute myeloid leukemia are considered AEs of special interest for rucaparib and other PARP inhibitors, no incidences of these AEs were reported.

**Table 2:** Most frequent (≥10% of patients) treatment-emergent adverse events of any grade in the safety population

TEAE	Overall (***N*** = 97)	
	Any grade, *n* (%)	Grade ≥3, *n* (%)
Asthenia/fatigue	56 (57.7)	8 (8.2)
Nausea	41 (42.3)	1 (1.0)
Anemia^a^	35 (36.1)	20 (20.6)
Decreased appetite	28 (28.9)	2 (2.1)
Thrombocytopenia^b^	22 (22.7)	11 (11.3)
Vomiting	22 (22.7)	1 (1.0)
Blood creatinine increased	21 (21.6)	1 (1.0)
Constipation	21 (21.6)	3 (3.1)
ALT/AST increased	17 (17.5)	5 (5.2)
Dysgeusia	16 (16.5)	0
Dyspnea	13 (13.4)	3 (3.1)
Weight decreased	13 (13.4)	0
Diarrhea	12 (12.4)	1 (1.0)
Urinary tract infection	12 (12.4)	4 (4.1)
Abdominal pain	11 (11.3)	1 (1.0)
Hypophosphatemia	10 (10.3)	5 (5.2)
Dehydration	10 (10.3)	2 (2.1)
Insomnia	10 (10.3)	0
Pyrexia	10 (10.3)	1 (1.0)

*ALT* alanine aminotransferase; *AST* aspartate aminotransferase; *TEAE* treatment-emergent adverse event.

Visit cutoff date: February 20, 2020.

^a^ Combined term for anemia or decreased hemoglobin.

^b^ Combined term for thrombocytopenia or decreased platelets.

Treatment was interrupted due to a TEAE for 50/97 (51.5%) patients, most frequently due to anemia (14/97 [14.4%]) and asthenia/fatigue (13/97 [13.4%]). Dose reduction due to a TEAE occurred in 23/97 (23.7%) patients, most commonly due to asthenia/fatigue (9/97 [9.3%]) and anemia (6/97 [6.2%]). Treatment discontinuation due to a TEAE (other than disease progression) occurred in 9/97 (9.3%) patients, with no specific TEAE reported in more than one patient. TEAEs other than disease progression resulted in death in three patients (3/97 [3.1%]): one each due to cardiac arrest, myocardial infarction, and respiratory failure. All three TEAEs were considered unrelated to rucaparib. Twenty additional deaths due to disease progression were reported.

### Pharmacokinetics

Mean (coefficient of variation) trough plasma concentration of rucaparib was 2130 ng/mL (86%; *n* = 47) at day 29, 1647 ng/mL (65%, *n* = 18) at day 57, and 2033 ng/mL (33%; *n* = 11) at day 85 (Supplementary Fig. S1).

### Genomic characteristics

To better understand the genomic features of this patient population with advanced UC, genomic profiling data was generated from 64 patients with adequate tumor tissue samples (see Supplementary Methods for detailed methodology for genomic analyses). Of those collected samples, 18.8% were from the sites of primary disease (bladder, renal pelvis, or ureter) and 81.2% were from the sites of local or distant metastases, the majority of which came from lymph node, liver, and lung. The median tumor mutational burden (TMB) was 6.3 mutations/megabase (*n* = 60) across the entire dataset, and all samples with known status were microsatellite stable (*n* = 59).

**Fig. 4:**
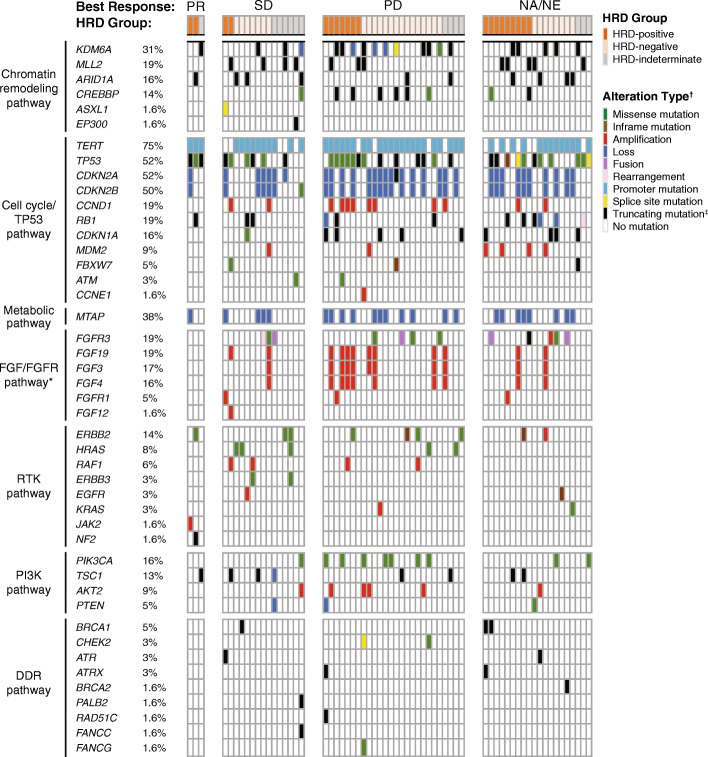
Genetic alterations in select pathways. Oncoprint generated from 64 tumor samples with sequencing data. Percentages shown indicate the deleterious gene alteration frequencies. *Only *FGFR1* and *FGFR3* alterations were identified; no *FGFR2* alterations were observed. ^†^Only one alteration type is presented when multiple alteration types were found within a single gene. ^‡^Includes truncating rearrangements. *DDR* DNA damage repair; *HRD* homologous recombination deficiency; *PD* progressive disease; *PR* partial response; *NA* not applicable; *NE* not evaluable; *SD* stable disease

Deleterious alterations in genes associated with cell cycle, fibroblast growth factor (FGF)/FGF receptor (FGFR), phosphatidylinositol-3-kinase (PI3K)/AKT, Ras/receptor tyrosine kinase (RTK), metabolic pathways, and chromatin remodeling pathways were frequently observed (Fig. 4). The most common genes with deleterious alterations within this patient population included the *TERT* promoter (75%), *CDKN2A* (52%), *TP53* (52%), *CDKN2B* (50%), *MTAP* (38%), *KDM6A* (31%), *CCND1* (19%), *FGF19* (19%), *FGFR3* (19%), *MLL2* (19%), and *RB1* (19%) (Fig. 4).

In an exploratory analysis, we assessed the frequency of alterations in 15 DDR genes (*ATM, BARD1, BRCA1, BRCA2, BRIP1, CDK12, CHEK2, FANCA, NBN, PALB2, RAD51, RAD51B, RAD51C, RAD51D*, *RAD54L*). In total, 10/64 (15.6%) patients had a deleterious alteration in one of these 15 genes (three *BRCA1,* two *ATM*, two *CHEK2*, one each for *BRCA2, PALB2*, *RAD51C;* Supplementary Table S2). Deleterious alterations in genes that are thought to be most strongly associated with PARP inhibitor sensitivity (*BRCA1*, *BRCA2*, *RAD51C*, *RAD51D*, *PALB2*) were observed in 6/64 (9.4%) patients (Supplementary Table S2). Alterations in these DDR genes were not associated with the antitumor activity of rucaparib for these patients. Additional genomic data, including zygosity and germline characteristics, and co-occurring alterations, are described in the Supplementary Results.

Interestingly, five patients provided genomic data from both recent and previously obtained archival tumor tissue samples that were acquired approximately 3 months to 2 years apart (Supplementary Table S3). In the paired samples, most of the deleterious genomic alterations were present in both samples. A substantial number of novel genomic alterations were observed in the recent specimen compared to the archival sample in only one of the five patients; the interval between the two sample collections was longest for this patient (763 days).

## Discussion

In the final analysis of ATLAS, although a number of patients with advanced UC had reductions in the sum of target lesions while receiving rucaparib, there were no confirmed radiographic responses. Notably, 45.4% of the patients had received two prior therapies for advanced UC. The safety and PK profile of rucaparib in patients with advanced UC were consistent with that observed in patients with ovarian, prostate, and other solid tumors [23, 30]. This suggests that the lack of observed efficacy was not likely due to changes in drug metabolism. Taken together, the results suggest that monotherapy treatment with rucaparib does not provide a meaningful benefit to unselected patients with previously-treated, advanced UC.

Among 50 ATLAS patients whose tumor tissue samples could be assessed for genomic LOH, 40% had HRD-positive tumors. Median percent genome-wide LOH of tumors from ATLAS patients was consistent with the data from TCGA-BLCA samples, suggesting that patients in this study were representative of a broader patient population with advanced UC in terms of their genomic characteristics despite the differences in the tumor sample tissue of origin (largely metastatic versus mostly primary), tumor sample heterogeneity, treatment history (one or two prior therapies versus chemotherapy-naive), and sequencing methodology (hybrid capture-based targeted gene panel versus whole-exome sequencing/single nucleotide polymorphism arrays). Many of the most frequently altered genes across the ATLAS samples also demonstrated substantial alteration frequency in the TCGA-BLCA dataset [26, 31], suggesting that many genomic alterations observed in the ATLAS patients may have occurred early in the evolution of UC. Similarly, the median TMB and microsatellite instability status of the ATLAS tumors were similar to those reported in other studies despite their inclusion of more heterogeneous samples [32, 33]. Deleterious alterations in DDR genes associated with increased sensitivity to PARP inhibitors in other tumor types (e.g. *BRCA1, BRCA2, PALB2, RAD51C* and *RAD51D*) were infrequent (9.4%) in ATLAS. This alteration frequency was similar to the frequencies in the TCGA-BLCA (7.9%) and in other reports in patients with UC (5.2–6.7%) [34, 35].

In clinical studies, rucaparib has shown antitumor activity in ovarian and prostate carcinomas with a deleterious germline or somatic *BRCA* mutation [24]. Rucaparib has also demonstrated benefit as switch maintenance treatment in patients with HRD-negative recurrent ovarian cancer [22]. However, no difference in response was observed among the HRD subgroups in our study, suggesting that tumor HRD status, as defined by genome-wide LOH, may not be a predictive biomarker of response for patients with metastatic UC. Given the small number of deleterious DDR gene alterations observed, there were insufficient data to determine a relationship between rucaparib activity and DDR gene alterations in metastatic UC. Prior research has suggested that sensitivity to PARP inhibition in patients with *BRCA* alterations may depend on the zygosity status of the alteration and/or tumor type [36]. Unfortunately, the significance of inactivating homozygous DDR gene alterations in metastatic UC for sensitivity to PARP inhibitor remains unclear because the majority of DDR gene alterations characterized in this study were heterozygous or the zygosity was unknown/not reported. The effect of rucaparib in multiple tumor types (including UC) with selected DDR gene alterations shown to be sensitive to PARP inhibition is being further evaluated in the LODESTAR trial (NCT04171700). Additionally, the effect of the PARP inhibitor olaparib for patients with metastatic UC with DNA-repair gene defects is being investigated in a phase 2 trial (NCT03375307).

Alterations in genes mapping to the cell cycle, *RTK*, *TP53*, *PI3K*, metabolic, and chromatin remodeling pathways were commonly seen in ATLAS patient samples, at frequencies similar to that in prior reports [31–33, 37]. Our data are in line with retrospective studies [38, 39] that assessed the landscape of genomic alterations in patients with UC who had similar characteristics to those in ATLAS. Alterations in cell cycle regulatory genes were also detected in the tumor DNA of patients enrolled in the phase Ib BISCAY trial, in which 15% of patients with UC carried an amplification in *RICTOR* or a deleterious alteration in *TSC1/TSC2* [37]. In a phase 2 study, genomic sequencing of archival tumor tissues from patients who developed advanced UC detected frequent alterations within *TP53* (52%), *CDKN2A/B* (34%), *ARID1A* (31%), and other cell cycle regulatory genes [40]. Actionable genetic alterations (e.g. *PIK3CA, ERBB2, FGFR3*) identified across many of these studies could possibly be targeted by various agents and may provide information about the specific outcomes of the treatment [26, 35, 39, 41]. Understanding more about the genomic landscape of advanced UC and the genomic characteristics of individual cases will aid in the development of putative biomarkers and therapeutic targets.

Since the initiation of the ATLAS study, several novel agents have been approved for use in the United States in patients with previously treated metastatic UC, including targeted agents, such as erdafitinib and antibody-drug conjugates, such as enfortumab vedotin [42, 43]; the targeted agent larotrectinib has also been approved for tissue-agnostic use in the United States and European Union [44, 45]. However, more treatment options are still needed for patients with metastatic UC, including those ineligible for any platinum therapy. Olaparib had previously reported cases of activity in a small set of patients with advanced UC and a DDR gene alteration [16, 17], while ATLAS enrolled a large unselected patient population. Recent studies have evaluated ICI in combination with PARP inhibitors with the aim of improving efficacy versus ICI or PARP inhibitor monotherapy. Responses have been observed in studies evaluating olaparib with durvalumab in patients with advanced UC: a response rate of 35.7% was observed among patients with DDR gene alterations in the BISCAY trial, and a pathologic complete response rate of 44.5% was seen in a single-arm phase 2 neoadjuvant trial [37, 46]. These data suggest that PARP inhibitors in combination with ICI may have antitumor activity in patients with UC. Evaluation of PARP inhibitor monotherapy and combination treatment for patients with UC is ongoing in other trials (NCT03459846, NCT03534492, NCT02546661, and EudraCT 2015–003249-25). The recent FDA approval of avelumab (with level I evidence) as switch maintenance treatment in patients with advanced UC with response or stable disease after PBC [47, 48] may also generate new opportunities for the evaluation of PARP inhibitors as combination therapy in this disease.

ATLAS is the largest study of PARP inhibitor treatment in patients with UC to-date. However, the study had several limitations. ATLAS was a single-arm, nonrandomized study with potential bias in patient selection and confounding factors associated with patient eligibility. For example, enrolled patients had advanced disease and most had received prior PBC, which could have reduced sensitivity to subsequent PARP inhibitor treatment. It should also be noted that patients were not selected based on genomic characteristics that may have differential sensitivities to treatment with a PARP inhibitor, such as tumor HRD status, alterations in DDR pathway genes, zygosity status of specific alterations, or germline alterations. Regarding the safety profile of rucaparib, while it was overall similar to that seen with rucaparib in other tumor types, median follow-up was limited to 2.7 months. Finally, although the study had a robust biomarker program to assess putative biomarkers of sensitivity to rucaparib, tumor tissue samples were not available from all enrolled patients. In addition, not all samples were sequenced successfully because of inadequate tumor content or volume, highlighting the challenges of genomic characterization. Future NGS-based genomic profiling of ctDNA samples collected from the patients in this study just prior to the start of treatment may be a complementary approach to identify contemporaneous genomic alterations that could impact outcomes of patients with this disease [49–51].

## Conclusions

Although rucaparib did not show confirmed responses in unselected patients with previously treated advanced UC, reductions in the sum of target lesions were observed in a number of patients. The safety profile of rucaparib in patients with advanced UC was consistent with that observed in patients with other solid tumors. Genomic profiling of tumor tissue samples has provided further insight into the molecular characterization of metastatic UC and showed that the median genome-wide LOH and deleterious genomic alterations in the ATLAS dataset were similar to the TCGA-BLCA dataset. Future studies should evaluate potential synergy of PARP inhibitors in combination with other therapies such as ICI, particularly in patients with metastatic UC associated with a DDR alteration.

## Supplementary Information

**Additional file 1: Supplementary Table S1.** Most Frequent (≥10% of Patients) Treatment-Related Adverse Events of Any Grade in the Safety Population. **Supplementary Table S2.** Summary of the Genetic Alterations in Tumor Tissue Samples and Tumor Responses in Patients With DDR Gene Mutation. **Supplementary Table S3.** Comparison of Genomic Characteristics From Archival and Recently Acquired Tumor Samples. **Supplementary Figure S1.** Time Profile for Mean (± Standard Deviation) Trough Plasma Concentration of Rucaparib

## Data Availability

All data generated or analysed during this study are included in this published article and its supplementary information files.

## Abbreviations

AE: adverse event
ALT: alanine aminotransferase
AST: aspartate aminotransferase
BRCA: BRCA1 or BRCA2
ctDNA: circulating tumor DNA
DCR: disease control rate
DDR: DNA damage repair
ECOG: Eastern Cooperative Oncology Group
ECOG PS: Eastern Cooperative Oncology Group performance status
FDA: Food and Drug Administration
FGF: fibroblast growth factor
FGFR: fibroblast growth factor receptor
HRD: homologous recombination deficiency
ICI: immune checkpoint inhibitor
IQR: interquartile range
ITT: intent-to-treat
LOH: loss of heterozygosity
NA: not applicable
NGS: next-generation sequencing
ORR: objective response rate
OS: overall survival
PARP: poly(ADP-ribose) polymerase
PBC: platinum-based chemotherapy
PD: progressive disease
PFS: progression-free survival
PI3K: phosphatidylinositol-3-kinase
PK: pharmacokinetic
PR: partial response
RECIST v1.1: Response Evaluation Criteria In Solid Tumors version 1.1
SD: stable disease
TCGA-BLCA: The Cancer Genome Atlas Urothelial Bladder Carcinoma
TEAE: treatment-emergent adverse event
TMB: tumor mutational burden
RTK: Ras/receptor tyrosine kinase
UC: urothelial carcinoma
