# Supplementary material for: Efficacy and safety of rucaparib in previously treated, locally advanced or metastatic urothelial carcinoma from a phase 2, open-label trial (ATLAS)
Source: BMC Cancer. 2021 May 24;21:593. doi: 10.1186/s12885-021-08085-z (PMC8147008; doi:10.1186/s12885-021-08085-z)

# Supplementary Information

ATLAS investigators……………………………………………………………………………………...39 Supplemental Methods………………………………………………………………………………...…..40 Additional inclusion criteria……………………………………………..……………………………40

Analysis of TCGA dataset………………………………………………………………………….…40

Dose reductions………………………………………………………………………………….…....40

Sample size calculation……………………………………………………………………………….40

Genomic analyses of patients’ tumor samples……………………………….........……………….…41

Significantly associated co-occurring alterations……………………………….………………….…42

Supplemental Results………………….………………………………………………….……………….42

Zygosity and germline characteristics…………………………………………………………...……42

Significantly associated co-occurring alterations……………………………………………….….…43

Supplementary References………………………………………………………………………..……….43

Supplemental Figures and Tables……………………………………….……………………………...…45

**Supplementary Table S1.** Most Frequent (≥10% of Patients) Treatment-Related Adverse Events of Any Grade in the Safety Population………………………………………………………………......45

**Supplementary Table S2.** Summary of the Genetic Alterations in Tumor Tissue Samples and Tumor Responses in Patients With DDR Gene Mutation………………………………………………….…46

**Supplementary Table S3.** Comparison of Genomic Characteristics From Archival and Recently Acquired Tumor Samples……………………………………………………………………..………48

**Supplementary Figure S1.** Time Profile for Mean (± Standard Deviation) Trough Plasma Concentration of Rucaparib………………………………………………………….………………..52

## ATLAS investigators

Y. Loriot (Institut Gustave Roussy), R. Morales-Barrera (Hospital Universitario Vall d' Hebron), M. Teo (Memorial Sloan Kettering Cancer Center), P. Grivas (Seattle Cancer Care Alliance), N. Vogelzang (Comprehensive Cancer Centers of Nevada), E. Grande (MD Anderson Cancer Center), N. Adra (Indiana University), A. Alva (University of Michigan), A. Necchi (Fondazione IRCCS Istituto Nazionale Tumori), Y. Zakharia (University of Iowa), D. Castellano (Hospital Universitario 12 de Octubre), G. Chatta (Roswell Park Comprehensive Cancer Institute), A. Drakaki (UCLA Department of Hematology/Oncology), S. Gupta (Huntsman Cancer Institute), A. Hussain (University of Maryland Greenebaum Cancer Center), S. Kochuparambil (Minnesota Oncology Hematology P.A. USO), R. McKay (UCSD Moores Cancer Center), D. Pouessel (Institut Claudius Regaud), A. Rodriguez-Vida (Hospital del Mar), P. Twardowski (John Wayne Cancer Institute), E. Uchio (Universityof California, Irvine), J. Vuky (Oregon Health and Science University), M. Boegemann (Universitatsklinikum Munster/Urologie und Kinderurologie), S. De Placido (Azienda Ospedaliera Universitaria Federico II Oncologia Medica), R. Dreicer (University of Virginia Health System), S. Feyerabend (Studienpraxis Urologie), R. Graham (UT/Erlanger Oncology & Hematology), S. Jain (Texas Oncology - Denton South USO - Denton, TX), A. Kalebasty (Norton Cancer Center), D. Kilari (Medical College of Wisconsin), J. Maroto Rey (Hospital Santa Creu i Sant Pau), M. Matrana (Ochsner Clinic Foundation), A. Morgans (Northwestern University), D. Morris (Urology Associates Clinical Research), P. Palyca (Lehigh Valley Health Network), J. Perez Gracia (Clinica Universitaria de Navarra Pamplona), F. Rolland (Institut de Cancerologie de l'Ouest Site Rene Gauducheau), I. Schnadig (USO - Northwest Cancer Specialists, P.C.), D. Shaffer (New York Oncology Hematology, P.C. USO - Albany, NY), N. Shore (Carolina Urologic Research Center)

## Supplementary Methods

**Additional inclusion criteria**

Eligible patients had histologically or cytologically confirmed locally advanced unresectable (tumor, node, metastasis [TNM] staging of T4b and any N; or any T and N2-3) or metastatic transitional cell carcinoma of the urothelium (including renal pelvis, ureter, urinary bladder, or urethra); those with mixed transitional/non-transitional cell histologies were allowed. No more than one prior platinum-based chemotherapy (PBC) was permitted for advanced disease. A patient was considered to have one prior PBC if there was a change of PBC within the same treatment regimen. A combination of PBC and radiation therapy was not considered as a prior PBC regimen.

**Analysis of TCGA dataset**

Data from the genomic analysis of TCGA urothelial bladder carcinoma dataset reported by Robertson et al. [1] was accessed and analyzed using Cbioportal [2, 3] online and custom in-house analysis tools.

**Dose reductions**

Dose reductions were permitted for patients in decrements of 100 mg if a grade ≥3 or inadequately controlled grade 2 adverse event was observed.

**Sample size calculation**

The overall sample size was determined by considering the number of patients needed for adequate safety and activity assessment of homologous recombination deficiency (HRD)-positive patients. Based on the estimated prevalence of 60% HRD-positive patients in this population, the study was designed to enroll approximately 200 patients, 120 of whom would be HRD-positive patients. The null hypothesis for response rate based on historical data in similar patient populations was *P* = 0.10. With a total of 200 patients, the study had greater than 90% power to reject the null hypothesis at a 5% significance level if the true response rate for rucaparib was 20%.

**Genomic analyses of patients’ tumor samples**

The data cutoff date for genomic analyses was January 14, 2020. Comprehensive genomic profiling was performed using a hybrid-capture based next-generation sequencing (NGS) approach to sequence a targeted panel of 310 cancer-related genes and to identify functionally classified gene alterations, including single nucleotide variants, short insertions/deletions, rearrangements, fusions, and copy number alterations as described in Frampton et al [4]. Zygosity and germline/somatic status of the alterations were computationally predicted based on a research–use only algorithm that has not been analytically validated [5]. An alteration in zygosity was determined by the loss of the second allele through loss of heterozygosity (LOH) at the locus of the alteration; other mechanisms that could lead to the loss of the other allele were not investigated. Genome-wide LOH was defined as the percentage of the interrogable genome with LOH and was evaluated using ~3500 single nucleotide polymorphisms as previously described [6, 7]. Genomic LOH values (for investigational use only) were reported in samples for which computationally derived tumor content was greater than 30%. Tumor mutational burden was defined as the number of mutations per megabase of DNA and was determined by measuring the number of synonymous and nonsynonymous somatic mutations occurring in the sequenced genes across 0.8–1.2 Mb genomic content as described [8]. Microsatellite status was determined by assessing the indel characteristics at 114 homopolymer repeat loci in or near the targeted gene regions analyzed as described. The genomic profiling analysis presented in this manuscript comprises one sample per patient even if multiple samples were provided; preference for inclusion was given to the most recently acquired tissue samples or those which returned genome-wide LOH results.

**Significantly associated co-occurring alterations**

Pairs of genes with deleterious alterations were assessed for their tendency to co-occur by quantifying how strongly the presence of one gene alteration was associated with the presence of another using the log2 odds ratio statistic. Co-occurring alterations that demonstrated a significant association were evaluated using one-sided Fisher’s exact test (*P* <0.05) and of Benjamini-Hochberg false discovery rate correction procedure (*q* <0.05).

## Supplementary Results

**Zygosity and germline characteristics**

Genomic profiling of the ATLAS samples using NGS revealed the genomic landscape of the ATLAS tumor samples by identifying single nucleotide variants, short insertions/deletions, rearrangements, fusions, and copy number alterations. Of particular interest were the deleterious DNA damage repair (DDR) gene alterations. Although zygosity and germline status were not reported for the gene rearrangement alterations, most of the identified DDR short variants were inferred to be heterozygous germline alterations. The only exceptions were two *ATM* missense alterations (one identified as homozygous and somatic, one as heterozygous and somatic), and the *RAD51C* frameshift alteration, characterized as somatic with unknown zygosity (Supplementary Table S2).

**Significantly associated co-occurring alterations**

Genomic analysis highlighted multiple co-occurring alterations in the ATLAS tumor samples. Co-occurring alterations that demonstrated a significant association included co-amplification of *FGF* genes (*FGF19* with *FGF3,* or *FGF4* and *FGF3* with *FGF4*); co-amplification of *CCND1* with *FGF3*, *FGF4*, or *FGF19;* co-amplification of *FGFR1* and *WHSCHL1*; co-deletion of *CDKN2A* with *CDKN2B*; and co-deletion of *CDKN2A* or *CDKN2B* with *MTAP* (Figure 4). These co-occurring copy number alterations were not expected due to the co-location within the genome of the gene pairs described.

## Supplementary References

1. Robertson AG, Kim J, Al-Ahmadie H, Bellmunt J, Guo G, Cherniack AD, et al. Comprehensive molecular characterization of muscle-invasive bladder cancer. Cell. 2017;171:540-556.

2. Cerami E, Gao J, Dogrusoz U, Gross BE, Sumer SO, Aksoy BA, et al. The cBio cancer genomics portal: an open platform for exploring multidimensional cancer genomics data. Cancer Discov. 2012;2:401-404.

3. Gao J, Aksoy BA, Dogrusoz U, Dresdner G, Gross B, Sumer SO, et al. Integrative analysis of complex cancer genomics and clinical profiles using the cBioPortal. Sci Signal. 2013;6:1-34.

4. Frampton GM, Fichtenholtz A, Otto GA, Wang K, Downing SR, He J, et al. Development and validation of a clinical cancer genomic profiling test based on massively parallel DNA sequencing. Nat Biotechnol. 2013;31:1023-1031.

5. Sun JX, He Y, Sanford E, Montesion M, Frampton GM, Vignot S, et al. A computational approach to distinguish somatic vs. germline origin of genomic alterations from deep sequencing of cancer specimens without a matched normal. PLoS Comput Biol. 2018;14:e1005965.

6. Coleman RL, Oza AM, Lorusso D, Aghajanian C, Oaknin A, Dean A, et al. Rucaparib maintenance treatment for recurrent ovarian carcinoma after response to platinum therapy (ARIEL3): a randomised, double-blind, placebo-controlled, phase 3 trial. Lancet. 2017;390:1949-1961.

7. Swisher EM, Lin KK, Oza AM, Scott CL, Giordano H, Sun J, et al. Rucaparib in relapsed, platinum-sensitive high-grade ovarian carcinoma (ARIEL2 Part 1): an international, multicentre, open-label, phase 2 trial. Lancet Oncol. 2017;18:75-87.

8. Chalmers ZR, Connelly CF, Fabrizio D, Gay L, Ali SM, Ennis R, et al. Analysis of 100,000 human cancer genomes reveals the landscape of tumor mutational burden. Genome Med. 2017;9:1-14

## Supplemental Figures and Tables

### **Supplementary Table S1** Most frequent (≥10% of patients) treatment-related adverse events of any grade in the safety population.

| **TRAE** | **Overall (*N* = 97)** | |
| --- | --- | --- |
|  |  |  |
|  | Any grade, *n* (%) | Grade ≥3, *n* (%) |
| Overall | 76 (78.4%) | 32 (33.0) |
| Asthenia/fatigue | 43 (44.3) | 6 (6.2) |
| Nausea | 35 (36.1) | 1 (1.0) |
| Anemia^a^ | 23 (23.7) | 13 (13.4) |
| Thrombocytopenia^b^ | 17 (17.5) | 8 (8.2) |
| Vomiting | 17 (17.5) | 1 (1.0) |
| ALT/AST increased | 14 (14.4) | 4 (4.1) |
| Decreased appetite | 14 (14.4) | 2 (2.1) |
| Blood creatinine increased | 12 (12.4) | 0 |
| Dysgeusia | 12 (12.4) | 0 |
| *ALT* alanine aminotransferase; *AST* aspartate aminotransferase; *TRAE* treatment-related adverse event.  Visit cutoff date: February 20, 2020.  ^a^ Combined term for anemia or decreased hemoglobin.  ^b^ Combined term for thrombocytopenia or decreased platelets. | | |

### **Supplementary Table S2** Summary of the genetic alterations in tumor tissue samples and tumor responses in patients with DDR gene mutation.^a^

| **Patient** | **Gene^a^** | **Deleterious alteration** | **Zygosity** | **Germline status^b^** | **% LOH** | **TMB** | **Best change % STL** |
| --- | --- | --- | --- | --- | --- | --- | --- |
| 1 | *ATM* | H2872R | Homozygous | Somatic | 16.8 | 6.3 | 48.9 |
| 2 | *ATM* | R337C | Heterozygous | Somatic | Indeterminate^d^ | 2.5 | –31.3 |
| 3 | *BRCA1* | V409fs*3 | Heterozygous | Germline | 9.9 | 13.9 | –7.8 |
| 4 | *BRCA1* | E23fs*17 | Heterozygous | Germline | 12.0 | 5.0 | NE^c^ |
| 5 | *CHEK2* | Splice site 1096-1G>A | Heterozygous | Germline | 7.2 | 25.2 | 50.0 |
| 6 | *CHEK2* | I157T | Heterozygous | Germline | 8.3 | 7.6 | 75.6 |
| 7 | *RAD51C* | E218fs*33 | Unknown^d^ | Somatic | 34.0 | 3.8 | 29.1 |
| 8 | *BRCA1* | Rearrangement (truncating) | Not reported^e^ | Not reported^e^ | 22.3 | 6.3 | NE^c^ |
| 9 | *BRCA2* | Rearrangement (truncating) | Not reported^e^ | Not reported^e^ | 5.6 | 6.3 | NE^c^ |
| 10 | *PALB2* | Rearrangement (truncating) | Not reported^e^ | Not reported^e^ | Indeterminate^d^ | 16.4 | 10.1 |
| *DDR* DNA damage repair; *LOH* loss of heterozygosity; *NE* not evaluable; *STL* sum of target lesions; *TMB* tumor mutational burden.  ^a^ Deleterious somatic and germline alterations in the following DDR genes are shown: *ATM, CHEK2*, *BRCA1*, *BRCA2*, *PALB2*, or *RAD51C*.  ^b^ Germline/somatic status was computationally predicted using a research–use only algorithm that has not been analytically validated.  ^c^ Not evaluable due to lack of data comparing baseline and posttreatment scans.  ^d^ Valid data could not be generated. ^e^Zygosity and germline/somatic status were not reported for gene rearrangements. | | | | | | | |

### **Supplementary Table S3** Comparison of genomic characteristics from archival and recently acquired tumor samples.

| **Patient** | **Archival specimen** | | | | **Recently acquired specimen^a^** | | | | Intervening therapy |
| --- | --- | --- | --- | --- | --- | --- | --- | --- | --- |
|  | Collection date | Tissue origin | Gene | Alteration | Collection date | Tissue origin | Gene | Alteration |  |
| 11 | Nov-23-2018 | Bladder | CREBBP | Q923* | Feb-27-2019 | Lymph node | CREBBP | Q923* | Cisplatin + gemcitabine |
|  |  |  | FGFR3 | S800fs*101 |  |  | FGFR3 | S800fs*101 |  |
|  |  |  | MLL2 | P480fs*450 |  |  | MLL2 | P480fs*450 |  |
|  |  |  | CDKN2A | loss |  |  | CDKN2A | loss |  |
|  |  |  | CDKN2B | loss |  |  | CDKN2B | loss |  |
|  |  |  | MDM2 | amp |  |  | MDM2 | amp |  |
|  |  |  | MTAP | loss |  |  | MTAP | loss |  |
| 12 | Nov-17-2017 | Bladder | TERT | promoter  -124C>T | Sep-5-2018 | Liver | TERT | promoter  −124C>T | Cisplatin + gemcitabine |
|  |  |  | TP53 | H179R |  |  | TP53 | H179R |  |
|  |  |  | MLL2 | Q3577fs*13 |  |  | MLL2 | Q3577fs*13 |  |
|  |  |  |  |  |  |  | RAD21 | splice site 274+1G>T |  |
|  |  |  | AKT2 | amp |  |  | AKT2 | amp |  |
|  |  |  | AXL | amp |  |  | AXL | amp |  |
|  |  |  | CCND1 | amp |  |  | CCND1 | amp |  |
|  |  |  | CDKN2A | loss |  |  | CDKN2A | loss |  |
|  |  |  | CDKN2B | loss |  |  | CDKN2B | loss |  |
|  |  |  | FGF19 | amp |  |  | FGF19 | amp |  |
|  |  |  | FGF3 | amp |  |  | FGF3 | amp |  |
|  |  |  | FGF4 | amp |  |  | FGF4 | amp |  |
|  |  |  | MTAP | loss |  |  | MTAP | loss |  |
| 13 | Dec-12-2017 | Renal pelvis | HRAS | Q61R | Feb-8-2019 | Psoas Muscle | HRAS | Q61R | Cisplatin + gemcitabine, atezolizumab |
|  |  |  | MERTK | D151V |  |  | MERTK | D151V |  |
|  |  |  | TERT | promoter  −124C>T |  |  | TERT | promoter  −124C>T |  |
|  |  |  | CDKN2A | loss |  |  | CDKN2A | loss |  |
|  |  |  | CDKN2B | loss |  |  | CDKN2B | loss |  |
|  |  |  | MTAP | loss |  |  | MTAP | loss |  |
| 14 | Jan-5-2018 | Lymph node | FGFR3 | S249C | Mar-11-2019 | Lymph node | FGFR3 | S249C | Cisplatin + gemcitabine, pembrolizumab |
|  |  |  | PIK3CA | Q546R |  |  | PIK3CA | Q546R |  |
|  |  |  | TERT | promoter  −124C>T |  |  | TERT | promoter  −124C>T |  |
|  |  |  | CDKN2A | loss |  |  | CDKN2A | loss |  |
|  |  |  | CDKN2B | loss |  |  | CDKN2B | loss |  |
|  |  |  | STAG2 | loss |  |  |  |  |  |
|  |  |  |  |  |  |  | HGF | amp |  |
| 15 | Oct-13-2016 | Kidney/ureter |  |  | Nov-15-2018 | Liver | KDM6A | W449* | Cisplatin + gemcitabine, pembrolizumab |
|  |  |  |  |  |  |  | SF3B1 | K666N |  |
|  |  |  | TERT | promoter  −124C>T |  |  | TERT | promoter  −124C>T |  |
|  |  |  | CCND1 | amp |  |  | CCND1 | amp |  |
|  |  |  | CDKN2A | loss |  |  | CDKN2A | loss |  |
|  |  |  | CDKN2B | loss |  |  | CDKN2B | loss |  |
|  |  |  | FGF10 | amp |  |  |  |  |  |
|  |  |  |  |  |  |  | ERBB2 | amp |  |
|  |  |  | FGF19 | amp |  |  | FGF19 | amp |  |
|  |  |  | FGF3 | amp |  |  | FGF3 | amp |  |
|  |  |  | FGF4 | amp |  |  | FGF4 | amp |  |
|  |  |  | MDM2 | amp |  |  | MDM2 | amp |  |
|  |  |  | RICTOR | amp |  |  |  |  |  |
|  |  |  | WHSC1L1 | amp |  |  |  |  |  |
|  |  |  | ZNF703 | amp |  |  |  |  |  |
| *amp* amplification; *loss* homozygous deletion; *HCl* hydrochloride.  Red font indicates the novel deleterious gene alterations identified between the two samples.  ^a^ Compulsory for participation in ATLAS. | | | | | | | | | |

### **Supplementary Fig. S1** Time profile for mean (± standard deviation) trough plasma concentration of rucaparib.


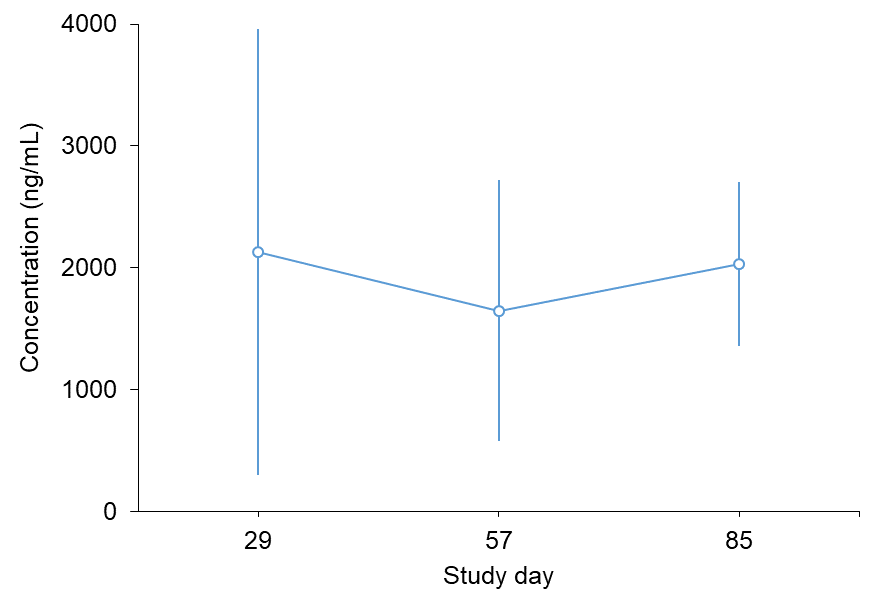

Supplement: Supplementary file 1 — Additional file 1: Supplementary Table S1. Most Frequent (≥10% of Patients) Treatment-Related Adverse Events of Any Grade in the Safety Population. Supplementary Table S2. Summary of the Genetic Alterations in Tumor Tissue Samples and Tumor Responses in Patients With DDR Gene Mutation. Supplementary Table S3. Comparison of Genomic Characteristics From Archival and Recently Acquired Tumor Samples. Supplementary Figure S1. Time Profile for Mean (± Standard Deviation) Trough Plasma Concentration of Rucaparib [file 12885_2021_8085_MOESM1_ESM.docx]
